# Applying Research to Public Health Questions: Timing and the Environmentally Relevant Dose

**DOI:** 10.1289/ehp.0901417

**Published:** 2009-11

**Authors:** Linda S. Birnbaum

**Affiliations:** Director, NIEHS and NTP, National Institutes of Health, Department of Health and Human Services, Research Triangle Park, North Carolina, E-mail: birnbaumls@niehs.nih.gov

The mission of National Institute of Environmental Health Sciences (NIEHS) is to improve the health of the American people by understanding the role of environmental exposures in disease and dysfunction. We accomplish this mission by conducting and funding research—including *in vitro*, animal, and human studies—on the health effects of environmental agents. Our goal is to prevent disease by identifying and reducing exposures to environmental agents that compromise health. It is clear that every complex disease has both an environmental and a genetic component. Thus, NIEHS-sponsored research must play an important role in understanding disease etiology. In the last few years there have been workshops (Melnick et al. 2002;vom Saal et al. 2007), manuscripts (Myers et al. 2009a, 2009b), and even society-position papers (The Endocrine Society 2009) indicating that increased use of environmental health science data by policy makers should lead to reductions in the human burden of disease.

There are several recent examples of how research supported by the NIEHS is leading to paradigm shifts in understanding how environmental toxicants—even at very low-level exposures—can have significant consequences, including dysfunction and disease. These paradigm shifts are being informed by new approaches for dose measurement. NIEHS researchers are turning their attention to the “environmentally relevant dose,” which is the dose in the range of typical human exposure as measured in tissue, blood, and urine of study subjects. Simply put, the environmentally relevant dose is based on the internal concentration of the toxicant rather than the administered dose.

In 2007, the NIEHS invited a panel of experts to Chapel Hill, North Carolina, for a scientific review of all literature published on bisphenol A (BPA). The expert panel then issued a consensus statement (vom Saal et al. 2007), which concluded that low environmentally relevant doses of BPA could cause numerous diseases in animal models, and that there was evidence for both low-dose effects and for nonmonotonic dose–response relationships. Overall, similar conclusions were reached by the National Toxicology Program’s Center for the Evaluation of Risks to Human Reproduction (NTP 2008), which focused on the developmental and reproductive effects of BPA.

An article in this issue of *Environmental Health Perspectives* (Myers et al. 2009b) highlights this discussion of low-dose effects and notes that nonmonotonic, or biphasic, dose–response curves are commonly observed in endocrinology. This suggests that high doses may not be appropriate to predict the safety of low doses when hormonally active or modulating compounds are studied. Their conclusions are supported by the position statement published by the Endocrine Society (2009). This debate—whether chemicals with endocrine-disrupting activity can cause toxicity at environmentally relevant doses—has been under way for more than a decade (Melnick et al. 2002). There are now low-dose data not only on BPA but also on phthalates, polychlorinated biphenyls (PCBs), dioxins, heavy metals such as lead and mercury, perchlorate, and some diverse pesticides such as hexachlorobenzene and atrazine. Indeed, the doses used in many animal toxicology studies result in internal concentrations that are in the range of human exposures.

Many of these low-dose studies demonstrate that the timing of exposure is critical to the outcome and that exposures during early life stages (fetal, infant, and pubertal) are particularly important. This recognition of critical windows of vulnerability not only demonstrates the developmental basis of disease but also that the timing, as well as the dose, makes the poison.

Understanding the connection between our health and our environment, with its mixture of chemicals, diet, and lifestyle stres-human genome; just as we have moved beyond “one gene, one disease,” we must move beyond “one chemical, one dose (range), one health outcome.” Reliability and validity are established in science by replication of findings in multiple independent studies. A weight-of-evidence approach is essential in understanding the public health impacts of environmental exposures.

## Figures and Tables

**Figure:**
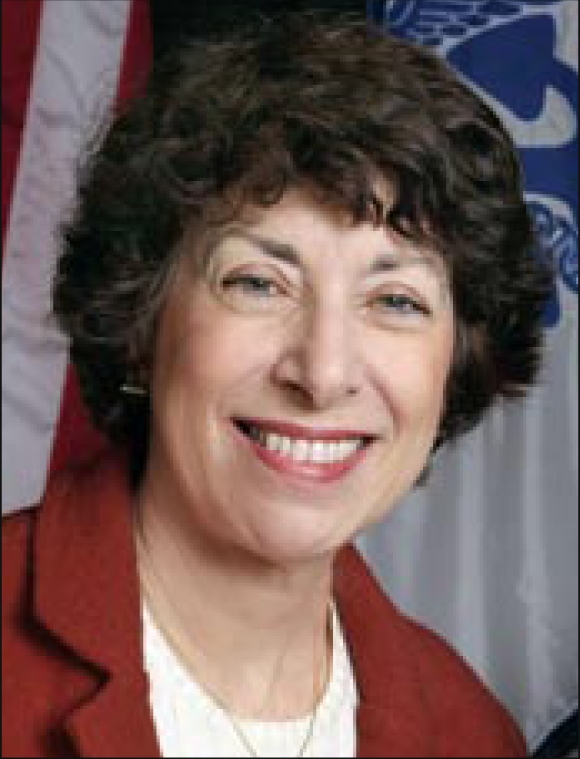
Linda S. Birnbaum
